# Unfolding the effects of decontamination treatments on the structural and functional integrity of N95 respirators via numerical simulations

**DOI:** 10.1038/s41598-022-08150-y

**Published:** 2022-03-09

**Authors:** Sumit Sharma, Fang Wang, P. V. Kameswara Rao, Ashwini K. Agrawal, Manjeet Jassal, Imre Szenti, Ákos Kukovecz, Amit Rawal, Ulf D. Schiller

**Affiliations:** 1 Department of Textile and Fibre Engineering, Indian Institute of Technology Delhi, Hauz Khas, New Delhi, India; 2 Department of Materials Science and Engineering, Clemson University, 161 Sirrine Hall, Clemson, SC 29634 USA; 3 Interdisciplinary Excellence Centre, Department of Applied and Environmental Chemistry, University of Szeged, Rerrich Béla tér 1, Szeged, 6720 Hungary

**Keywords:** Theory and computation, Imaging techniques

## Abstract

Filtering facepiece respirators (FFRs) provide effective protection against diseases spread through airborne infectious droplets and particles. The widespread use of FFRs during the COVID-19 pandemic has not only led to supply shortages, but the disposal of single-use facemasks also threatens the environment with a new kind of plastic pollution. While limited reuse of filtering facepiece respirators has been permitted as a crisis capacity strategy, there are currently no standard test methods available for decontamination before their repeated use. The decontamination of respirators can compromise the structural and functional integrity by reducing the filtration efficiency and breathability. Digital segmentation of X-ray microcomputed tomography (microCT) scans of the meltblown nonwoven layers of a specific N95 respirator model (Venus-4400) after treatment with one and five cycles of liquid hydrogen peroxide, ultraviolet radiation, moist heat, and aqueous soap solution enabled us to perform filtration simulations of decontaminated respirators. The computed filtration efficiencies for 0.3 µm particles agreed well with experimental measurements, and the distribution of particle penetration depths was correlated with the structural changes resulting from decontamination. The combination of X-ray microCT imaging with numerical simulations thus provides a strategy for quantitative evaluation of the effectiveness of decontamination treatments for a specific respirator model.

## Introduction

The coronavirus disease 2019 (COVID-19), triggered by severe acute respiratory syndrome coronavirus-2 (SARS-CoV-2), epitomizes the pandemic of the century that resembles the spread and severity of the 1918 influenza epidemic^1^. This highly contagious virus poses a severe risk to the general public and, in particular, to healthcare workers. At the heart of the unprecedented COVID-19 crisis, healthcare workers face challenges in treating patients and developing short-term and long-term strategies to reduce the spread of infection^2^. To ensure the safety of healthcare workers, it is essential to have sufficient supplies of personal protective equipment (PPE), including respirators, body protection equipment, facial protection, gloves, shoe covers, head, and neck cover^3^. N95 respirators limit the passage of airborne droplets by blocking at least 95% of very small (0.3 μm) test particles^4–6^. The use of N95 respirators has been recommended for healthcare workers while caring for COVID-19 patients, specifically when performing procedures such as intubation, extubation, non-invasive ventilation, etc., which generate high concentrations of aerosols^7^. Globally, the unprecedented demand for the N95 respirators has not only led to demand-supply gaps, but extraordinary efforts have been directed in utilizing homemade cloth masks to match the filtration performance^8^. However, a plethora of methodological and analytical problems were later reported, and it was suggested that the commonly used fabrics should not be conflated with the N95 respirators in terms of filtration efficiency^9–11^. Limited reuse of N95 respirators after decontamination has been considered as a crisis capacity strategy. Dry heat, steam, UV, detergent, chemicals (H_2_O_2_, ethanol, isopropanol, bleach, chlorine dioxide) have been employed as decontamination strategies for N95 respirators and melt-blown fabrics^12–23^. However, disinfecting treatments can affect the structural and functional integrity of the filtering material. Therefore, the effectiveness of disinfecting treatments methods has to be evaluated with respect to filtration performance after each decontamination cycle.

With advanced imaging techniques, it is now possible to obtain three-dimensional (3D) images of micro-and nano-structured materials with high resolution. However, the accurate characterization of material properties based on images remains challenging due to noise and artifacts in the raw images. Careful image processing and segmentation are necessary for accurate predictions of filtration characteristics, which subsequently assist in simulating the digitally reconstructed fibrous media. In the past, the Digital Materials Laboratory Software (GeoDict®) has been successfully employed to determine the microstructural characteristics and filtration performance of various kinds of filter media, including nonwoven materials. For instance, Azimian et al.^24^ modeled variations of filter media for oil filtration to optimize the fiber volume distribution to increase the dust holding capacity while maintaining a low-pressure drop of the filter element. In addition, the flow and efficiency simulations of aerosol filtration in fibrous media were carried out based on synchrotron X-ray images and found that the results were in good agreement with experimental measurements^25,26^. Wang et al.^27^ investigated the dependency of filtration efficiency and pressure drop on the filter thickness. They reported an exponential relationship for the filter efficiency and a linear relationship for the pressure drop. On the other hand, Wang et al.^28^ performed simulations of polyacrylonitrile membranes and observed the change of the particle deposition pattern from surface filtration to deep bed filtration. Maddineni et al.^29^ used a numerical approach to investigate the effect of collision and adhesion parameters on the capture efficiency of aerosol particles in fibrous media. In particular, they studied particle bounce and re-entrainment mechanisms and found that the Hamaker adhesion model yielded a good agreement with experimental data for the capture efficiency. Their results suggested that flow re-entrainment can increase particle penetration, and the effect depends mainly on the fiber volume fraction. Bai et al.^30^ constructed a layered filter model based on measured fiber dimensions and fiber orientation distributions. They demonstrated that the simulations of the filter efficiency for the digital twin model reproduced the measured values for a real sample. GeoDict has been used in the past to uncover the filtration performance of electret filter media based on their morphology and other physical characteristics without considering the effect of electrostatic charges^31–33^. Further, GeoDict has successfully generated virtual fibrous media with varying solid volume fraction and orientation distribution and performed simulations to study the dependence of permeability on the microstructural characteristics^34,35^. In a comprehensive review of theoretical filtration models, Bai et al.^36^ compared theoretical, simulated, and experimental data for different filtration mechanisms, including diffusion, interception, and impaction. These previous works show that numerical simulations can be a valuable tool for predicting the filtration performance of nonwoven materials.

The central aim of this work was to perform numerical simulations of the particle filtration process in the meltblown nonwoven layers of a specific N95 respirator before and after decontaminating with one and five cycles of liquid hydrogen peroxide, ultraviolet radiation, moist heat, aqueous soap solution at a face velocity (~ 3.89 m/s) that emulates speaking conditions^37^. Specifically, this research work provides insights into the effect of local structural heterogeneities developed after decontamination treatments on the filtration performance of the meltblown layers of N95 respirators. The unavoidable gaps between experiments and simulations stem from the inability to account for the electrostatic charges in the meltblown layers, the shape of the droplets, various interactions, and other uncertainties^31^. The input geometries for the numerical simulations were obtained from X-ray microCT scans, as described in our previous work^38^. Here, we imported the stack of X-ray microCT images into the GeoDict software package to perform numerical simulations of the airflow and particle trajectories through the nonwoven layers.

## Results

**Figure 1:**
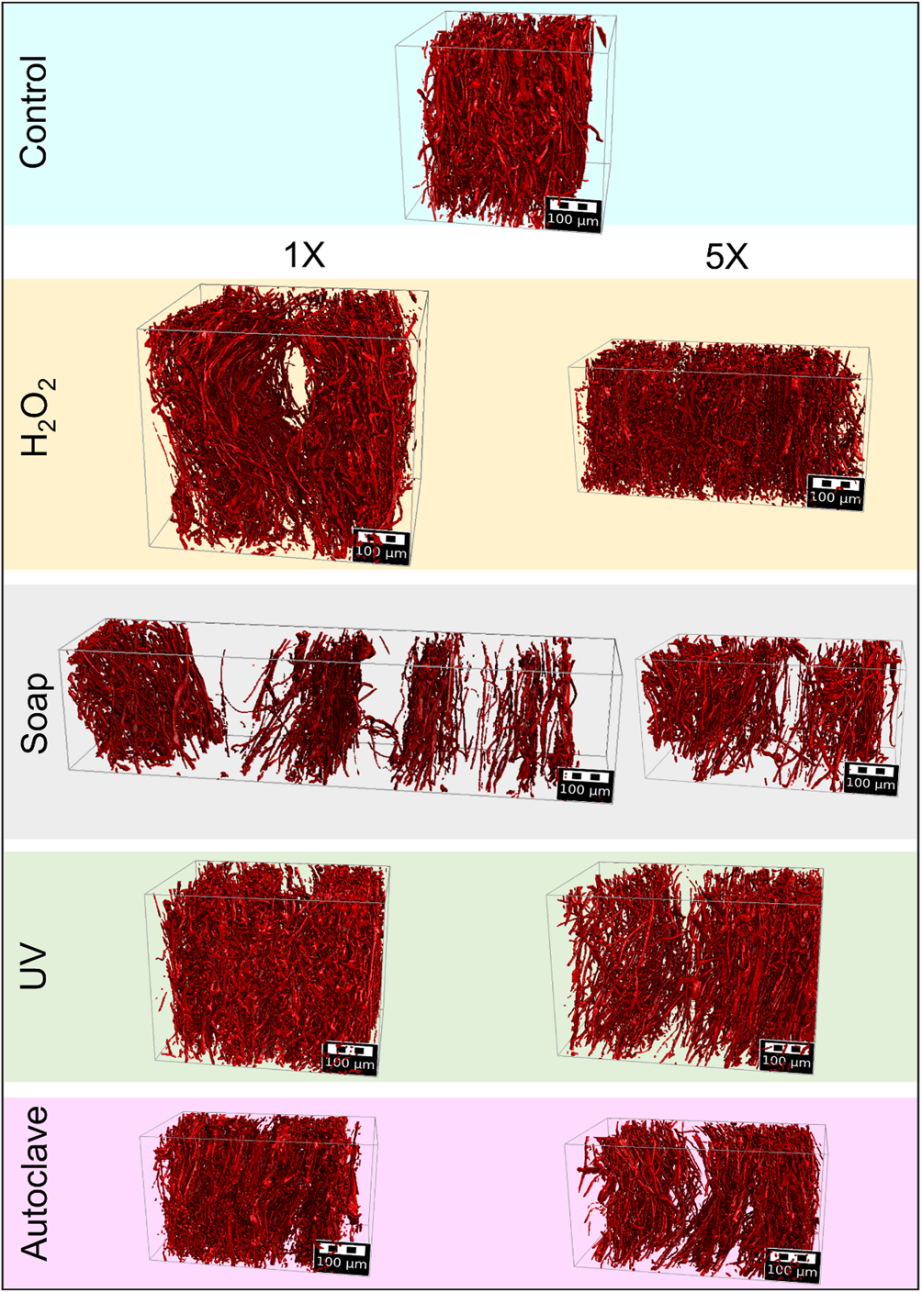
Digital segmentation of X-ray microCT images of meltblown layers of N95 respirators before (control) and after one and five cycles of H_2_O_2_, soap, UV, and autoclave treatments.

A commercially available electret-based Venus-4400 disposable N95 flat-fold respirator was decontaminated with one and five cycles of treatment with aqueous hydrogen peroxide (H_2_O_2_), aqueous soap solution, autoclaving, and ultraviolet (UV) light. The detailed decontamination treatments and subsequent X-ray microCT analysis are reported elsewhere^38^. Here, we utilized the X-ray microCT image stacks to generate digital twins of the meltblown layers of N95 respirators. The 3D geometries resulting from digital segmentation are shown in Fig. 1. The polypropylene meltblown layers made up of fibers with a mean diameter of 4.8 µm had a thickness of 0.58 mm, mass per unit area of 45.91 g/m^2^, and porosity of 91%^38^.

Filtration simulations were performed with two different particle collision models—‘caught on first touch’ and Hamaker model—which allowed us to investigate the role of adhesion interactions. The “caught on first touch” collision model provided an upper bound for the filtration efficiency. In the Hamaker model, the simulated filtration efficiency depended on the Hamaker constant and the restitution coefficient, as shown in Supplementary Fig. S1a and S1b. The model parameters were calibrated for the control sample, and the same parameters were used for the simulations of the decontaminated samples. The sample IDs were assigned based on the type of treatment (H_2_O_2_ is hydrogen peroxide, SO is soap, UV is ultraviolet light, and AU is autoclave) and the number of cycles. For example, UV-5X refers to the sample that was treated with five cycles of ultraviolet radiation treatment. The flow field and distribution of captured particles within a subregion of the control sample are shown in Supplementary Fig. S1c and S1d. The particle trajectories of 0.3 µm particles in the control sample obtained by numerical simulations with the ‘caught on first touch’ and the Hamaker model are illustrated in Fig. S1e and S1f. The Supplementary Videos S1–S3 show the trajectories of particles as they propagate through the filtration layer.

The flow fields and distributions of captured particles in selected subregions of the decontaminated samples after one treatment cycle are shown in Supplementary Fig. S2. As discussed below, the flow fields showed considerable variation due to structural changes within the nonwovens. The distribution of captured particles also varied considerably between treatment methods which affected the filtration efficiency accordingly. Supplementary Table S1 shows the predicted filtration efficiency for all simulated subregions stratified by treatment method and the number of cycles. The distribution of the penetration depth of 0.3 µm particles (obtained from filtration simulations using the Hamaker model) in control and decontaminated samples are shown in Supplementary Fig. S3 for all selected subregions.

**Figure 2:**
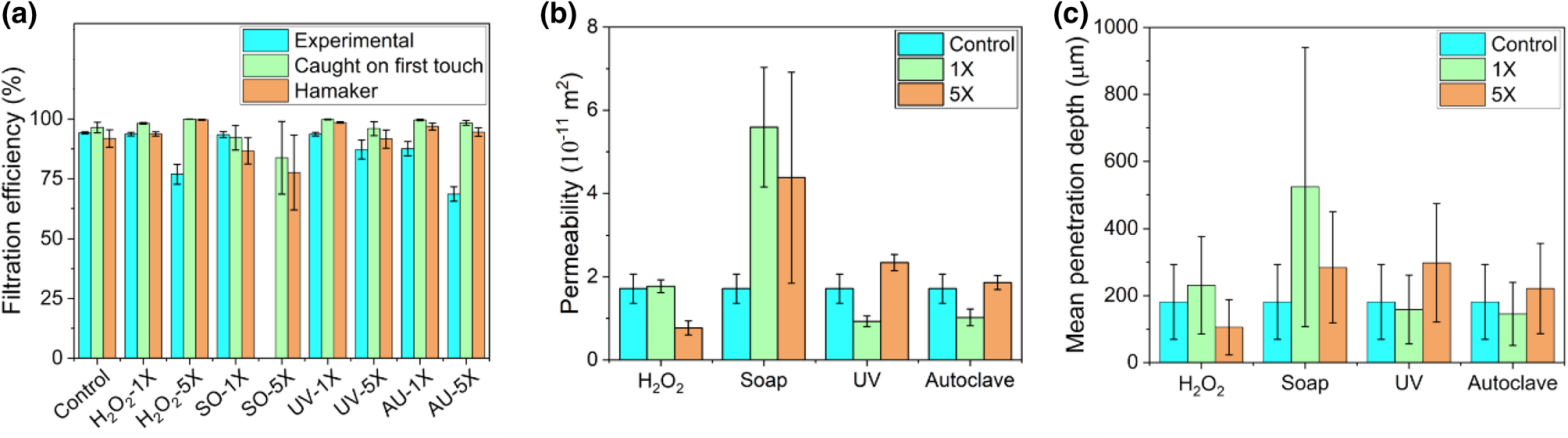
Comparison of (**a**) filtration efficiency of N95 respirators after various decontamination treatments. ‘Experimental’ refers to the experimentally obtained filtration efficiency of the control and decontaminated N95 respirators obtained from Ref. 38. The filtration performance after five cycles of soap treatment could not be determined experimentally due to a very high pressure drop. Analysis of individual layers of the N95 respirator revealed that the high-pressure drop was likely due to the entrapment of soap particles in the meltblown layers. (**b**) Comparison of the permeability of the nonwoven layers of N95 respirators before and after decontamination treatments. (**c**) Comparison of the depth of particle penetration into the nonwoven layers of N95 respirators before and after decontamination treatments.

The comparison between numerical simulations and experimental measurements is shown in Fig. 2. The filtration efficiency (Fig. 2a) agreed well with the experimental findings; however, the predicted values tended to be higher than the experimental measurements. Figure 2b, c show the permeability and mean particle penetration depth obtained from the numerical simulations. The control sample’s permeability (Fig. 2b) was in the range of values reported for N95 masks elsewhere in the literature^39^. For H_2_O_2_ treatment, the permeability remained unaltered after one treatment cycle and decreased after five treatment cycles. For soap treatment, the permeability increased significantly after treatment due to the disintegration of the nonwoven layers. The variation between different subregions was larger for soap treatment than other treatment methods. The permeability decreased after one cycle of UV and heat treatment but remained comparable to the control sample after five treatment cycles. This can be associated with the degradation and subsequent compaction of the fibrous structure of the nonwoven layers^38^.

The mean particle penetration depth (Fig. 2c) for 0.3 µm particles increased after decontamination treatments. For solution-based treatments, this occurred after the first treatment cycle, while for UV and heat treatment, the increase became noticeable after five cycles.

### Effect of H_2_O_2_ decontamination treatment

**Figure 3:**
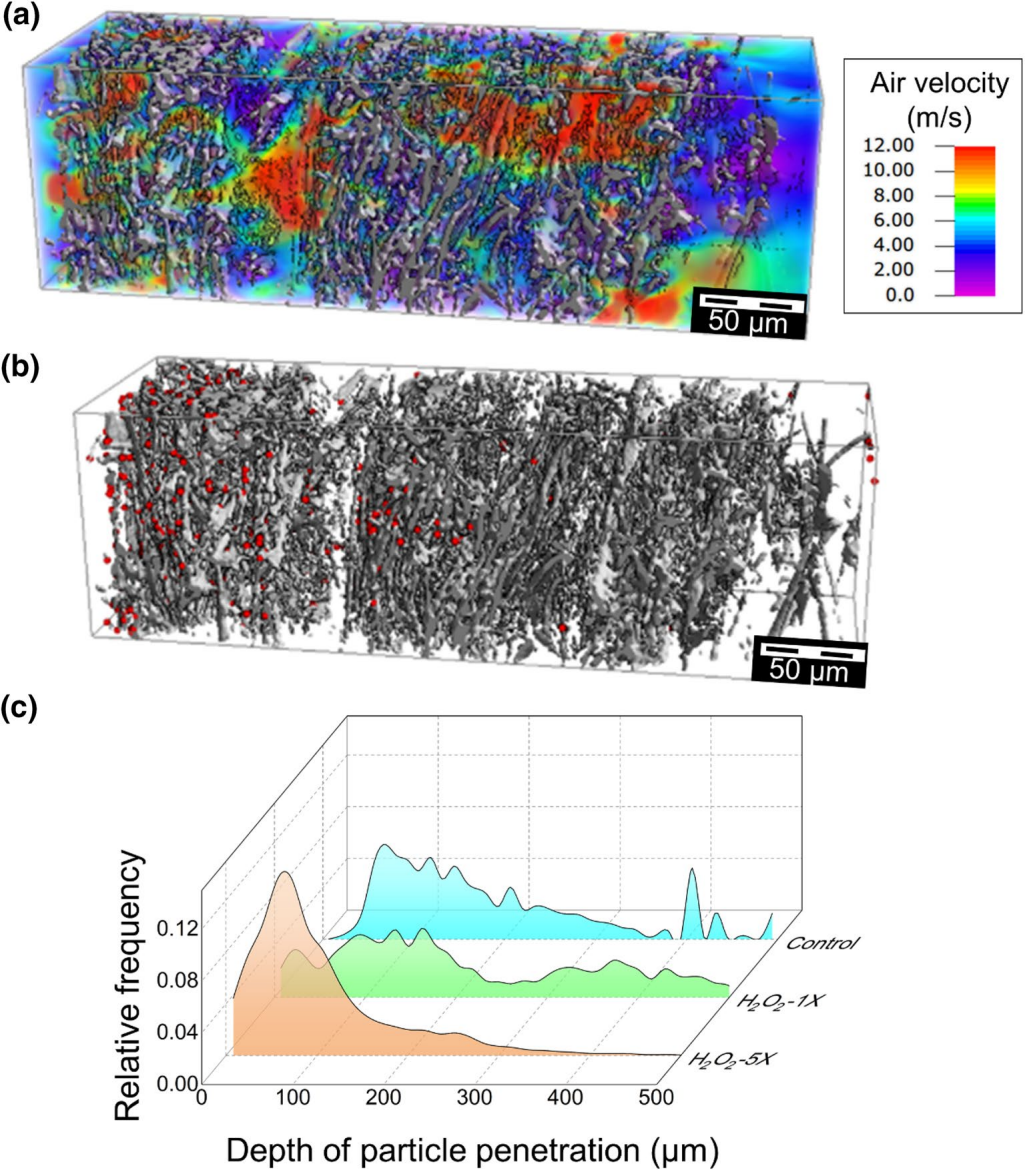
Elucidating the role of aqueous H_2_O_2_ treatment on (**a**) flow field and (**b**) distribution of captured particles for selected subregion after five cycles of treatment. (**c**) Comparison of the particle distribution across the depth of the control sample after one and five cycles. The displayed subregion has a thickness of 640 µm, a porosity of 0.90, resulting in a filtration efficiency of 99.5% after five cycles of H_2_O_2_ treatment.

The X-ray microCT analysis reported that after the first cycle of H_2_O_2_ treatment, voids were created in the meltblown layers that increased the local pore sizes^38^. The voids were clearly observed in the digital image segmentation (see Fig. 1); however, the calculated porosity of a subregion that contains such a void remained unaltered (see H2O2-1X Subregion 5 in Supplementary Table S1). Figure 3a, b show the flow field and distribution of captured particles in a selected subregion after five treatment cycles. The average filtration efficiency was 93.8% after one cycle and 99.7% after five cycles of H_2_O_2_ treatment. The distribution of particle penetration depths obtained from filtration simulations using the Hamaker model is shown in Fig. 3c. After one treatment cycle, the mean penetration depth of 0.3 µm particles increased to 231 µm. After five cycles of treatment, more particles were captured at the upstream side of the filtration layer, and the mean particle penetration depth decreased to 106 µm.

### Effect of soap decontamination treatment

**Figure 4:**
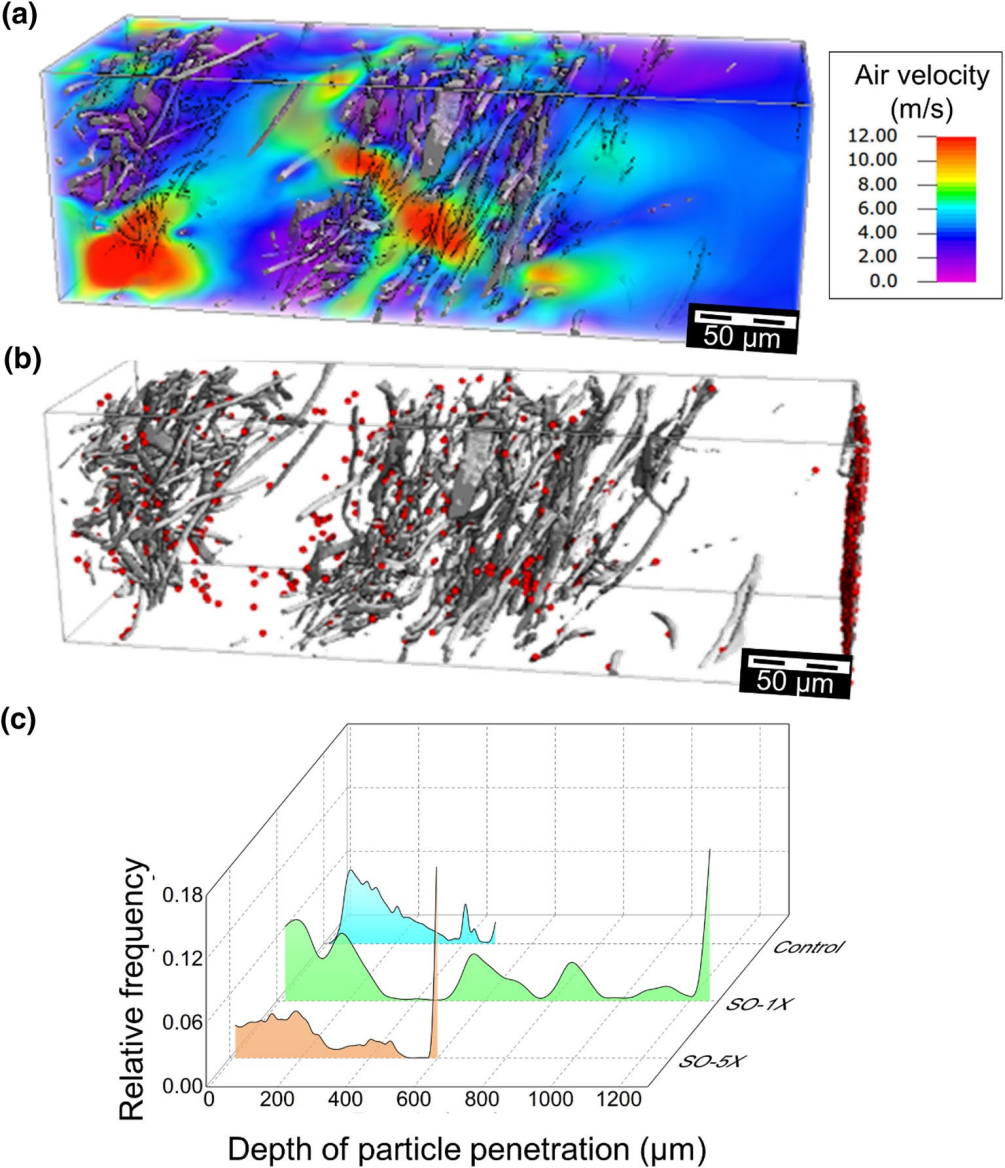
Elucidating the role of aqueous soap treatment on (**a**) Flow field and (**b**) Distribution of captured particles for selected subregion after five treatment cycles. (**c**) Comparison of the particle distribution across the depth of the control sample after one and five cycles. The displayed subregion has a thickness of 600 µm, a porosity of 0.97, resulting in a filtration efficiency of 63.9% after five cycles of soap treatment.

Soap decontamination treatment led to a substantial disintegration of the filtration layers. The flow field (Fig. 4a) in different subregions of the sample was susceptible to the severity of structural damage and the thickness of separated layers. This led to a considerable variation of the filtration efficiency in different subregions. The filtration efficiency obtained was as low as 80% and 60.4% after one and five cycles, respectively (see Supplementary Table S1). Further, the sample disintegration caused a substantial rise in the mean penetration depth of particles. The distribution of captured particles (Fig. 4b) showed large variations between sample subregions. The distribution of particle penetration depths (Fig. 4c) obtained from filtration simulations using the Hamaker model yielded a mean penetration depth of 524 µm after one cycle and 284 µm after five cycles of soap treatment. A greater number of particles penetrated through the disintegrated structure, reducing the average filtration efficiency to 86.7% after one cycle and 77.6% after five cycles of soap treatment.

### Effect of UV decontamination treatment

**Figure 5:**
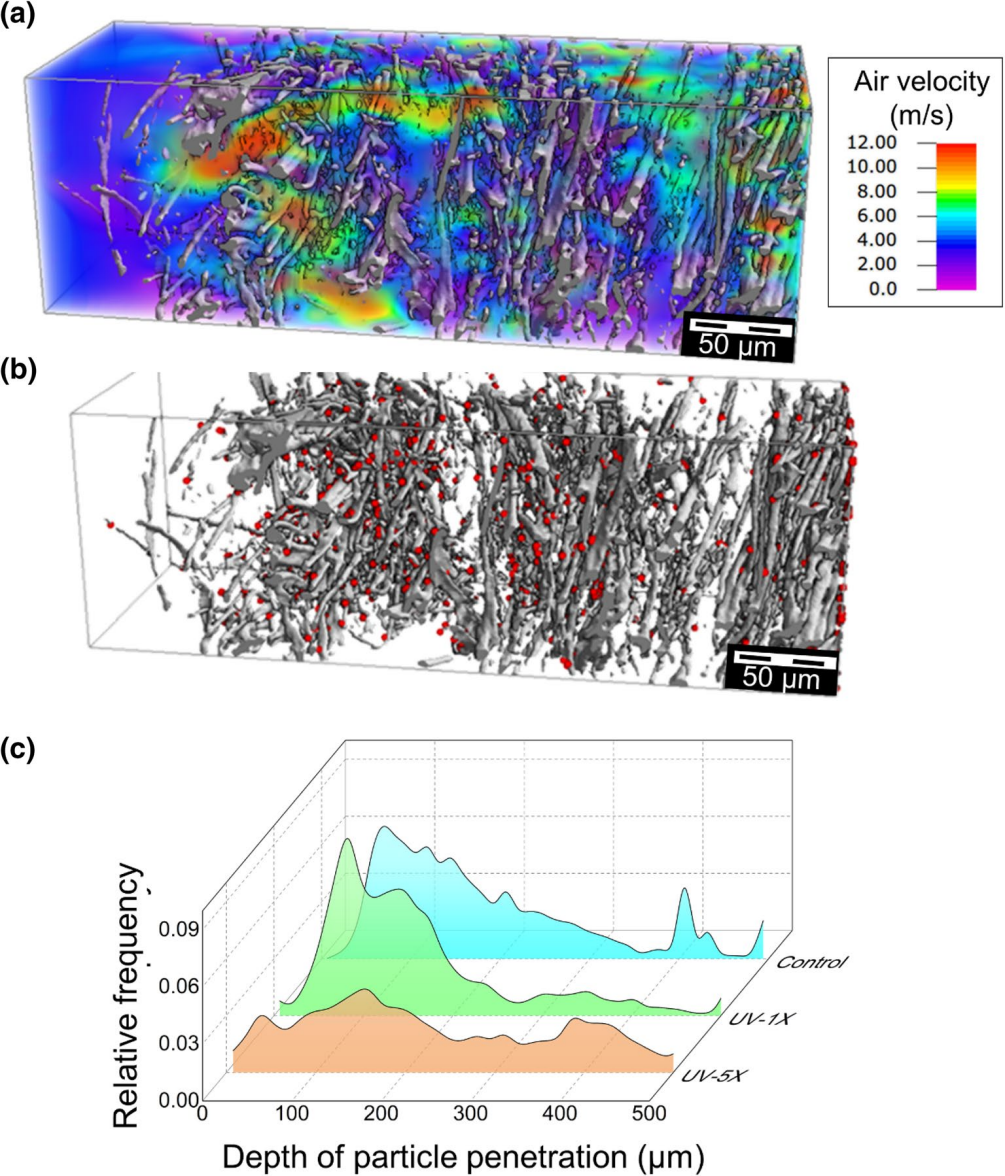
Elucidating the role of UV treatment on (**a**) Flow field and (**b**) Distribution of captured particles for the selected subregion after five treatment cycles. (**c**) Comparison of the particle distribution across the depth of the control sample after one and five cycles. The displayed subregion has a thickness of 600 µm, a porosity of 0.94, resulting in a filtration efficiency of 86.2% after five cycles of UV treatment.

X-ray microCT analysis revealed a slight reduction in porosity for UV-treated samples due to narrower pores^38^. Figure 5 shows the flow field and distribution of captured particles in a selected subregion after five cycles of UV treatment. The flow field (Fig. 5a) through the disintegrated sample showed considerable variability of the local velocity magnitude. The distribution of captured particles (Fig. 5b) spanned the whole thickness of the sample. The distribution of particle penetration depths is shown in Fig. 5c. The mean penetration depth of 0.3 µm particles (obtained from filtration simulations using the Hamaker model) increased to 159 µm after one cycle, and 298 µm after five cycles of UV treatment. The average filtration efficiency was 98.6% after one cycle and 91.6% after five cycles of UV treatment. While the computed filtration efficiency was slightly higher than the measured value, it reproduced the observed trend for a higher number of treatment cycles.

### Effect of autoclave decontamination treatment

**Figure 6:**
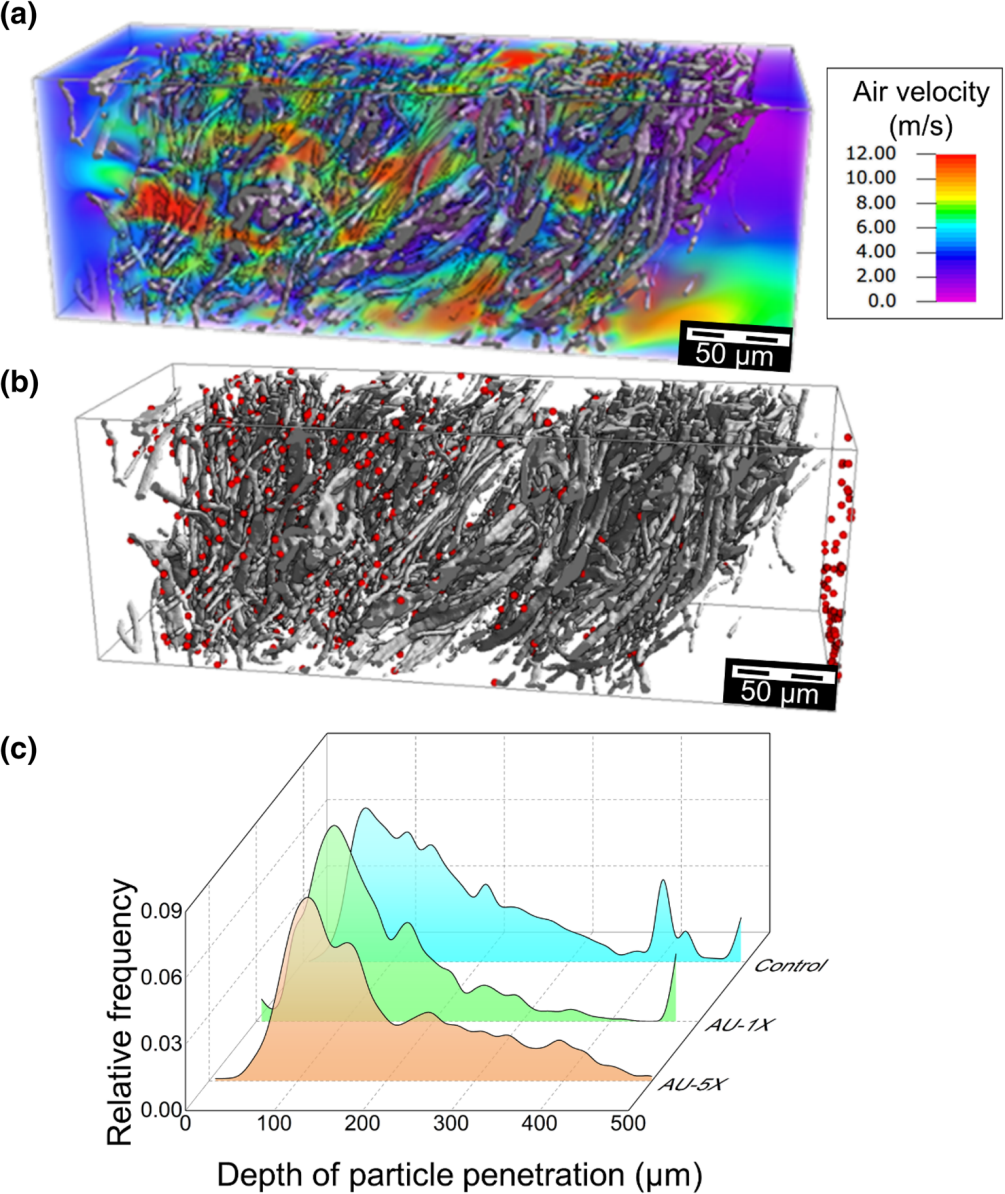
Elucidating the role of autoclave treatment on (**a**) Flow field and (**b**) Distribution of captured particles for selected subregion after five treatment cycles. (**c**) Comparison of the particle distribution across the depth of the control sample after one and five cycles. The displayed subregion has a thickness of 560 µm, a porosity of 0.92, resulting in a filtration efficiency of 93.6% after five cycles of autoclave treatment.

The X-ray microCT analysis of the autoclaved samples indicated minimal structural changes in the meltblown layers^38^. However, the digital segmentation revealed compaction of the layers and re-alignment of fibers. The flow field (Fig. 6a) contained regions of higher velocity, which increased impaction and captured the particles towards the inflow side of the sample. Greater variability in the mean penetration depth of particles was also observed, where the distribution of captured particles (Fig. 6b) extended across the entire thickness. The distribution of particle penetration depths (Fig. 6c) obtained from filtration simulations using the Hamaker model yielded a mean penetration depth of 146 µm after one cycle and 221 µm after five cycles of autoclaving. The average filtration efficiency was 96.9% after one cycle and 94.5% after five heat treatment cycles. The predicted values were higher than the measured filtration efficiency, and the trend for a higher number of cycles was weak.

## Discussion

The present study deals with the numerical simulations of airflow and particle transport in the key filtration layers of meltblown nonwoven layers of a commercially available N95 respirator (Venus-4400 disposable N95 flat-fold respirator) after one and five cycles of decontamination with liquid hydrogen peroxide, ultraviolet radiation, moist heat, and aqueous soap solution at a face velocity (~ 3.89 m/s) that emulates speaking conditions. The input geometries were obtained from X-ray microCT scans, and the simulation model parameters were calibrated for an untreated control sample. To elucidate the effect of the structural heterogeneity on the filtration efficiency of the treated samples, we investigated the flow field and distribution of captured particles within subregions of the digitized samples. In particular, we analyzed how far individual particles penetrated the nonwoven layers before they were caught on a fiber surface.

The analysis of five subregions of each sample revealed that structural heterogeneity correlates with considerable variability in the local airflow, particle penetration, and filtration efficiency. For instance, we observed that the effect of structural voids in H_2_O_2_ treated samples is offset by compaction of the surrounding fibers such that the overall filtration efficiency is retained. The penetration depth increased after the first cycle for the solution-based treatments, while the difference before and after one cycle was insignificant for the UV and heat treatments. However, an increase in the penetration depth was observed after five treatment cycles for the latter. Further, the variation of the filtration efficiency between different subregions increased with the number of cycles, particularly for soap treatment. These observations suggest that the *local* structural heterogeneity of the meltblown layers has a crucial impact on the overall filtration efficiency of N95 respirators. In particular, variation of filtration efficiency across the surface of the respirator is a cause of concern, as regions of low filtration efficiency can compromise the functional integrity. It is thus essential to assess the entire filtration layer of decontaminated respirators.

Whereas the filtration efficiency predicted by the simulations generally agreed well with the trends observed in experiments, the simulated values tended to be higher than the experimental measurement, most notably after five H_2_O_2_ and heat treatment cycles. This suggests that besides structural changes, the decontamination treatments can alter the physico-chemical properties of the filter material. For H_2_O_2_ and heat-treated samples, the changes of pore sizes and fiber orientation in the nonwoven layers may not affect the filtration efficiency as strongly as the surface adhesion properties of the fibers. When investigating the influence of the interaction parameters of the Hamaker model on the simulated filtration efficiency of the control sample, we found that the filtration efficiency was sensitive to both the Hamaker constant and the restitution coefficient. These parameters were tuned to match the filtration efficiency of the control sample and thus, could not capture quantitatively the changes in surface interactions caused by the treatments. Therefore, a deviation of the predicted filtration efficiency from the measured value was inevitable. Future research should focus on a detailed investigation of the adhesion properties after decontamination. Our simulations provided some insights into the potential effect of decontamination treatments on adhesion interactions but their effect on the pressure drop still needs to be investigated in the future. Nevertheless, the relatively good agreement between the predicted and measured filtration efficiency of the UV treated samples indicated that UV treatment did not change the surface adhesion as strongly as heat or H_2_O_2_ treatment.

Broadly speaking, our results indicate that solution-based treatments are less effective at preserving the structural and functional integrity of the meltblown nonwoven filtration layers of N95 respirators. Although the soap treatment is highly convenient for mask decontamination, our findings demonstrate that the filtration performance of the N95 respirator can be considerably reduced, as also reported elsewhere in the literature^20,23^. For UV- and heat-treated samples, the numerical results confirmed the experimental observation that the filtration efficiency deteriorated less severely with a higher number of treatment cycles. It is worth noting that we have not considered several aspects that are also relevant to respirator performance after decontamination, such as virus inactivation, off-gassing of decontamination chemicals, or fit performance. These factors should be evaluated along with filtration performance when considering respirator reuse.

As aforementioned, we analyzed the filtration efficiency for particles of 0.3 µm at a face velocity of 3.89 m/s that emulates speaking conditions. At this relatively high velocity, the deposition efficiency for diffusive trapping is significantly reduced due to the high Peclet number. Under these conditions, the Stokes number is also high, and therefore impaction and interception are the dominating mechanisms for particle deposition. This is corroborated by the observation that most particles are captured near the inflow surface of the nonwoven layers. The collision models in GeoDict invariably assume that particles remain entrapped by attaching to fiber, and there is no re-entrainment. However, re-entrainment is more likely to occur for agglomerated particle clusters. This would lead to a shift of the distribution towards larger particle sizes that are unlikely to penetrate the whole thickness of the nonwoven layers. Therefore, we consider a negligible effect of re-entrainment on the count of 0.3 µm particles in the experiments and simulations. We note that the particle transport model used herein does not consider breakup or evaporation that may play a role in aerosol filtration. As refined models for respiratory droplets and saliva plumes are emerging^40,41^, it will be possible to include the interactions of droplets with fibrous materials in filtration simulations. To this end, our integrated experimental and simulation approach provides a systematic strategy for quantitative evaluation of the filtration efficiency of decontaminated filtering facepiece respirators. Future scope of work can include the effect of the electrostatic charges in the electret meltblown layers of the N95 respirators.

## Methods

### Digital segmentation of X-ray microCT images

Digital twins of the experimental samples were created with the help of Digital Materials Laboratory Software GeoDict. The raw X-ray microCT images were imported into GeoDict using the ‘ImportGeo-Vol’ module. The images were aligned such that the fiber layers were oriented perpendicular to the Z-axis. The imported volume images were then cropped to the volume-of-interest (VOI), where fibers were present across the whole thickness so that the calculated porosity was not affected by void regions at the borders of the experimental image. The cropped volume was further processed by applying a denoising filter and a non-local means filter to remove speckle noise. The denoising was modulated by three parameters, a patch radius that determined the size of edges, a search window radius that defined the neighborhood around each voxel, and the filter strength that controlled the amount of smoothing. The filtered images were segmented using a threshold segmentation where the threshold was automatically determined using Otsu’s method^42^. The parameters for the image processing and segmentation in GeoDict are given in Supplementary Table S2. Following the segmentation, the binary structure was further processed by removing unconnected objects smaller than 500 voxels. The properties of the resulting structure were analyzed using the ‘FiberFind’ module in GeoDict.

To account for the structural inhomogeneity of the treated samples, we analyzed five different subregions of each sample. The subregions were chosen by visual inspection of the digitized X-ray microCT images to represent areas of varying thickness, degree of delamination, and distortion of the layers of the samples. The domain sizes of the subregions within the original image are specified in Supplementary Table S1 of the supplemental material.

### Numerical simulation of air flow and particle filtration

The airflow through the digital samples was simulated with the ‘FlowDict’ module in GeoDict. The air was considered a viscous and incompressible fluid described by the steady-state mass and momentum conservation equations. Therefore,

1 $$\nabla \cdot \overrightarrow{u}=0,$$

2 $$\rho \left(\overrightarrow{u}\cdot \nabla \overrightarrow{u}\right)=-\nabla p+\mu {\nabla }^{2}\overrightarrow{u},$$

where *ρ* denotes the fluid density, $\overrightarrow{u}$ is the flow velocity, *p* is the scalar pressure, and *µ* is the dynamic viscosity.

At a low Reynolds number, the momentum equation can be approximated by the Stokes equation,

3 $$-\nabla p+\mu {\nabla }^{2}\overrightarrow{u}=0.$$

The numerical simulations of particle filtration were performed using the ‘FlowDict’ module of GeoDict. The simple, fast Fourier transform solver (SimpleFFT) was employed to obtain the flow field, where velocity boundary conditions were used at the inlet to prescribe the mean face velocity in the z-direction. An error-bound stopping criterion was used to compare the computed permeability to the extrapolated value from previous time steps and stop the solver if the relative difference fell below 0.001. The parameters for the flow and filtration simulation in FlowDict are given in Supplementary Table S3.

### Numerical simulation of particle filtration

The flow field was then used to calculate the filtration efficiency with the ‘FilterDict’ module. The Lagrangian particle tracking algorithm in GeoDict assumes a dilute particle concentration such that particle collisions can be neglected and the advected particles do not perturb the flow field. The trajectory of the particles is described by the following stochastic equations,

$${m}_{p}\frac{d{\overrightarrow{v_{p}}}}{dt}=3\pi \mu \frac{{d}_{p}}{Cu}\left(\overrightarrow{u}-{\overrightarrow{v_{p}}}+\sqrt{\frac{2{k}_{B}T}{\gamma }}\frac{d\overrightarrow{W}}{dt}\right),$$

4 $$\frac{d{\overrightarrow{r_{p}}}}{dt}={\overrightarrow{v_{p}}}$$

where ${\overrightarrow{r_{p}}}$ denotes the particle position, ${\overrightarrow{v_{p}}}$ is the particle velocity, ${m}_{p}$ is the particle mass, ${d}_{p}$ is the particle diameter, $\gamma$ is the friction coefficient, ${k}_{B}T$ is the thermal energy, and $d\overrightarrow{W}$ is a stochastic Wiener process that generates thermal fluctuations, i.e., Brownian motion.

For sub-micron particles, a slip effect was taken into account via Cunningham slip factor $Cu$ given by

5 $$Cu=1+\frac{\lambda }{{d}_{p}}\left[2.34+1.05 \mathrm{exp}\left(-0.39\frac{{d}_{p}}{\lambda }\right)\right],$$

where λ is the mean free path of air.

Here, the particles were initially placed at a distance of 1 $\mu m$ from the boundary of an inflow domain that was automatically determined by GeoDict. The size distribution of the particles was specified according to experimental conditions (see Supplementary Table S4). The ‘Filtration efficiency’ module of GeoDict was used to determine the overall filtration efficiency of the meltblown layers and the fractional filter efficiency for each given particle size. The filtration efficiency for a single batch of particles was calculated using different collision models^24,43^. The ‘caught on first touch’ model assumes that a particle is captured upon the first contact with a fiber surface and yields an upper limit for the filtration efficiency. In addition, we used the Hamaker model to account for the adhesion forces between particles and surfaces. The model considers van der Waals interactions as the dominant forces, which can be expressed as^44,45^,

6 $${F}_{vdW}=\frac{A{d}_{p}}{12{h}^{2}},$$

where $h$ denotes the distance between the particle and the fiber surface, and $A$ is the Hamaker constant modeling the strength of the surface adhesion.

A particle colliding with the fiber surface is considered captured if the kinetic energy is small compared to the adhesion energy, in case the velocity is sufficiently small, i.e.,

7 $${v}^{2}<\frac{A}{\pi \rho h{d}_{p}^{2}},$$

where $\rho$ is the particle density. In case the kinetic energy exceeds the adhesion energy, the particle is not considered captured and loses a fraction of kinetic energy according to a restitution coefficient that defines the ratio of the velocities after and before the collision.

For dust particles interacting with polypropylene fibers in the air, a universal value for the Hamaker constant is difficult to determine due to the heterogeneous composition of the dust. Izadi et al.^46^ reported the Hamaker constant for PMMA/silica and PDMS/silica contact in a dry condition to be 6.4 × 10^−20^ J and 5.5 × 10^−20^ J, respectively. Pan et al.^47^ calculated the Hamaker constant A = 6.67 × 10^−21^ J for PET fiber interacting with solid particles. For organic molecules interacting in the air, the Hamaker constants have been found^48^ in the range of (4–7) × 10^−20^ J. Based on these values, we used an estimated value of 5.5 × 10^−20^ J as the Hamaker constant in our numerical simulations.

The restitution coefficient was determined by fitting the simulation results with experimental measurements for the control sample. For a restitution coefficient of 0.1, the simulated filtration efficiency of the control sample matched with the experimental data reasonably well. In previous studies, Pan et al.^47^ and Maddineni et al.^29^ used a similar restitution coefficient value.

All simulations were carried out on Clemson University’s Palmetto cluster using 8 Intel Xeon 6148G parallel CPU cores. Each flow simulation was completed within a time span of 1.5–30 h, depending on the computational domain size.

## Supplementary Information

Supplementary Information.

Supplementary Video 1.

Supplementary Video 2.

Supplementary Video 3.

## Data Availability

The datasets generated during and analyzed during the current study are available from the corresponding author on reasonable request. Correspondence and requests for materials should be addressed to AR and UDS.
