# Supplementary material for: Unfolding the effects of decontamination treatments on the structural and functional integrity of N95 respirators via numerical simulations
Source: Sci Rep. 2022 Mar 9;12:4191. doi: 10.1038/s41598-022-08150-y (PMC8906365; doi:10.1038/s41598-022-08150-y)
Supplement: Supplementary file 1 — Supplementary Information. [file 41598_2022_8150_MOESM1_ESM.docx]

**Unfolding the effects of decontamination treatments on the structural and functional integrity of N95 respirators via numerical simulations**

**Sumit Sharma**^a^**, Fang Wang**^b^**, P.V. Kameswara Rao**^a^**, Ashwini K. Agrawal**^a^**, Manjeet Jassal**^a^**, Imre Szenti**^c^**, Ákos Kukovecz**^c^**, Amit Rawal**^a^**, Ulf D. Schiller**^b^

*^a^Department of Textile and Fibre Engineering, Indian Institute of Technology Delhi, Hauz Khas, New Delhi, India*

^b^Department of Materials Science and Engineering, Clemson University, 161 Sirrine Hall, Clemson, SC 29634, USA

^c^Interdisciplinary Excellence Centre, Department of Applied and Environmental Chemistry, University of Szeged, H-6720, Rerrich Béla tér 1, Szeged, Hungary

# **Supplementary Material**

1. **Videos**
2. **Tables**
3. **Figures**
4. **Videos**

**Video S1:** Particle trajectories during the filtration simulation of the control sample using the “caught on first touch” collision model in GeoDict are depicted. The numerical simulation has been performed in a domain of size $200\times200\times400 \mu m$. Only 0.3 $\mu m$ particles are displayed in the video, and the particle diameter is enlarged 20 times for visualization purposes. The video shows the particle motion over 191 $\mu s$ in real-time.

**Video S2:** Particle trajectories during the filtration simulation of the control sample using the Hamaker collision model in GeoDict are depicted. The numerical simulation has been performed in a domain of size $200\times200\times400 \mu m$. Only 0.3 $\mu m$ particles are displayed in the video, and the particle diameter is enlarged 20 times for visualization purposes. The video shows the particle motion over 210 $\mu s$ in real-time.

**Video S3:** Motion of a single particle (diameter ~ 0.3 $\mu m$) in a control sample is followed during the filtration simulation by setting the Hamaker collision model in GeoDict. The particle diameter is enlarged 20 times for visualization purposes. The video also shows all particles with different diameters in the fiber layer as well.

1. **Tables**

**Table S1.** Porosity and filtration efficiency of different sub-regions simulated using GeoDict.

| Sample | Sub-region | Domain size ($\boldsymbol{\mu m}$) | Threshold | Porosity | Overall Porosity | Filtration Efficiency (%) |
| --- | --- | --- | --- | --- | --- | --- |
| Control | 1 | 400$\times$400$\times$400 | 30.5 | 0.92 | 0.90 | 91.5 |
|  | 2 | 200$\times$200$\times$400 |  | 0.93 |  | 92.0 |
|  | 3 | 200$\times$200$\times$400 |  | 0.91 |  | 96.8 |
|  | 4 | 200$\times$200$\times$400 |  | 0.93 |  | 86.6 |
|  | 5 | 200$\times$200$\times$400 |  | 0.90 |  | 92.6 |
|  |  |  |  |  |  |  |
| H_2_O_2_-1X | 1 | 500$\times$500$\times$550 | 41.5 | 0.90 | 0.92 | 93.6 |
|  | 2 | 200$\times$200$\times$550 |  | 0.91 |  | 93.5 |
|  | 3 | 200$\times$200$\times$550 |  | 0.92 |  | 95.0 |
|  | 4 | 200$\times$200$\times$550 |  | 0.91 |  | 92.7 |
|  | 5 | 200$\times$200$\times$550 |  | 0.93 |  | 94.2 |
|  |  |  |  |  |  |  |
| H_2_O_2_-5X | 1 | 400$\times$400$\times$400 | 24.5 | 0.89 | 0.87 | 99.7 |
|  | 2 | 200$\times$200$\times$400 |  | 0.90 |  | 99.9 |
|  | 3 | 200$\times$200$\times$400 |  | 0.90 |  | 99.5 |
|  | 4 | 200$\times$200$\times$400 |  | 0.89 |  | 99.9 |
|  | 5 | 200$\times$200$\times$400 |  | 0.90 |  | 99.6 |
|  |  |  |  |  |  |  |
| UV-1X | 1 | 500$\times$500$\times$550 | 41.5 | 0.89 | 0.87 | 99.0 |
|  | 2 | 200$\times$200$\times$550 |  | 0.88 |  | 98.2 |
|  | 3 | 200$\times$200$\times$550 |  | 0.89 |  | 98.5 |
|  | 4 | 200$\times$200$\times$550 |  | 0.88 |  | 98.6 |
|  | 5 | 200$\times$200$\times$550 |  | 0.90 |  | 98.7 |
|  |  |  |  |  |  |  |
| UV-5X | 1 | 400$\times$400$\times$400 | 24.5 | 0.94 | 0.91 | 89.9 |
|  | 2 | 200$\times$200$\times$400 |  | 0.94 |  | 86.2 |
|  | 3 | 200$\times$200$\times$400 |  | 0.95 |  | 96.2 |

(Continued)

| Sample | Sub-region | Domain size ($\boldsymbol{\mu m}$) | Threshold | Porosity | Overall Porosity | Filtration Efficiency (%) | |
| --- | --- | --- | --- | --- | --- | --- | --- |
|  | 4 | 200$\times$200$\times$400 |  | 0.93 |  | | 92.2 |
|  | 5 | 200$\times$200$\times$400 |  | 0.93 |  | | 93.7 |
|  |  |  |  |  |  | |  |
| SO-1X | 1 | 500$\times$500$\times$550 | 41.5 | 0.96 | 0.96 | | 90.6 |
|  | 2 | 200$\times$200$\times$550 |  | 0.96 |  |  | 80.0 |
|  | 3 | 200$\times$200$\times$550 |  | 0.95 |  |  | 93.5 |
|  | 4 | 200$\times$200$\times$550 |  | 0.96 |  |  | 83.3 |
|  | 5 | 200$\times$200$\times$550 |  | 0.96 |  |  | 86.0 |
|  |  |  |  |  |  | |  |
| SO-5X | 1 | 400$\times$400$\times$400 | 24.5 | 0.95 | 0.95 | | 77.0 |
|  | 2 | 200$\times$200$\times$400 |  | 0.95 |  |  | 60.4 |
|  | 3 | 200$\times$200$\times$400 |  | 0.94 |  |  | 93.7 |
|  | 4 | 200$\times$200$\times$400 |  | 0.97 |  |  | 97.3 |
|  | 5 | 200$\times$200$\times$400 |  | 0.94 |  |  | 93.1 |
|  |  |  |  |  |  | |  |
| AU-1X | 1 | 300$\times$300$\times$460 | 33.5 | 0.88 | 0.86 | | 96.8 |
|  | 2 | 200$\times$200$\times$460 |  | 0.89 |  |  | 97.9 |
|  | 3 | 200$\times$200$\times$460 |  | 0.90 |  |  | 96.2 |
|  | 4 | 200$\times$200$\times$460 |  | 0.87 |  |  | 98.7 |
|  | 5 | 200$\times$200$\times$460 |  | 0.91 |  |  | 95.0 |
|  |  |  |  |  |  | |  |
| AU-5X | 1 | 400$\times$400$\times$400 | 24.5 | 0.93 | 0.92 | | 96.4 |
|  | 2 | 200$\times$200$\times$400 |  | 0.92 |  |  | 96.2 |
|  | 3 | 200$\times$200$\times$400 |  | 0.94 |  |  | 94.2 |
|  | 4 | 200$\times$200$\times$400 |  | 0.92 |  |  | 93.6 |
|  | 5 | 200$\times$200$\times$400 |  | 0.93 |  |  | 92.3 |

**Table S2.** Parameters used in the image segmentation process.

| **Process** | **Parameter** |  |
| --- | --- | --- |
| Denoising  (Non-local means filter) | Patch radius | 1 voxel |
|  | Search window radius | 3 voxels |
|  | Strength | 0.5 |
| Segmentation  (Otsu single threshold) | Threshold value | Varied |
| Cleansing | Number of Voxels | 500 |

**Table S3.** Model parameters used for the simulation of airflow and particle filtration.

| **Parameters** | **Units** | **Values** |
| --- | --- | --- |
| Fluid | - | Air |
| Test temperature | $℃$ | 20 |
| Fluid density | $kg/m^{3}$ | 1.204 |
| Fluid dynamic viscosity | $kg/ms$ | 1.834 $\times{10}^{-5}$ |
| Flow motion | - | Creeping flow |
| Mean flow velocity | m/s | 3.89 |
| Slip length | M | 0 |
| Particle motion simulated | - | Brownian |
| Cunningham correction (Mean free path) | M | 6.6 $\times{10}^{-8}$ |
| Reflect particles at inflow plane | - | Yes |
| Particle charge | - | No charge |
| Collison model | - | Caught on first touch/Hamaker |
| Particle density | $kg/m^{3}$ | 2650 |
| Collision diameter | µm | Same as particle diameter |
| Flow Solver | - | SimpleFFT |
| Error bound | - | 0.001 |
| Velocity/Pressure relaxation | - | 0.5, 0.5 |

**Table S4.** Size distribution of particles in the airflow. The number of particles flowing per minute was measured experimentally, and the corresponding percentage was used to generate the same size distribution for the simulations.

| **Particle diameter (**$\boldsymbol{\mu m}$**)** | **No. of particles flowing per minute** | **Count (%)** |
| --- | --- | --- |
| 0.3 | 1958107 | 81.4 |
| 0.5 | 384840 | 16.0 |
| 1 | 57162 | 2.4 |
| 3 | 3107 | 0.1 |
| 5 | 668 | 0.03 |

1. **Figures**

*
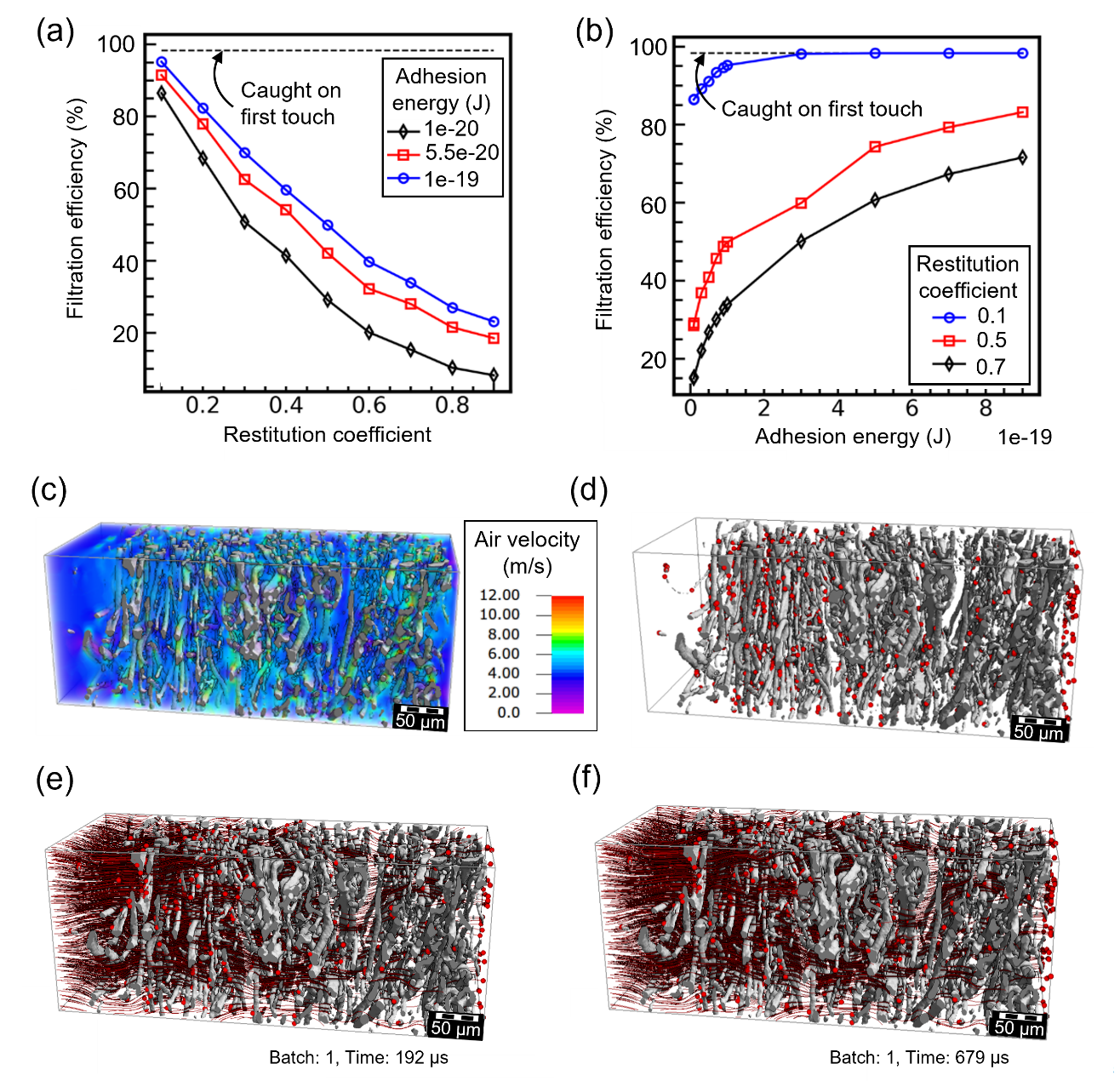
*

**Figure S1.** Effect of model parameters (a) Adhesion energy and (b) Restitution coefficient on the filtration efficiency of the control sample. (c) Flow field and (d) distribution of captured particles for selected subregions of the control sample. The displayed subregion has a thickness of 400 µm, porosity of 0.93 that resulted in a filtration efficiency of 92% after five cycles of H_2_O_2_ treatment. Visualization of 0.3 $\mu m$ particle trajectories in air flow obtained by numerical simulations in the control sample with (e) the ‘caught on first touch’ model and (f) the Hamaker model. The drawn particles are enlarged by a scale factor of 20 for visualization purposes.


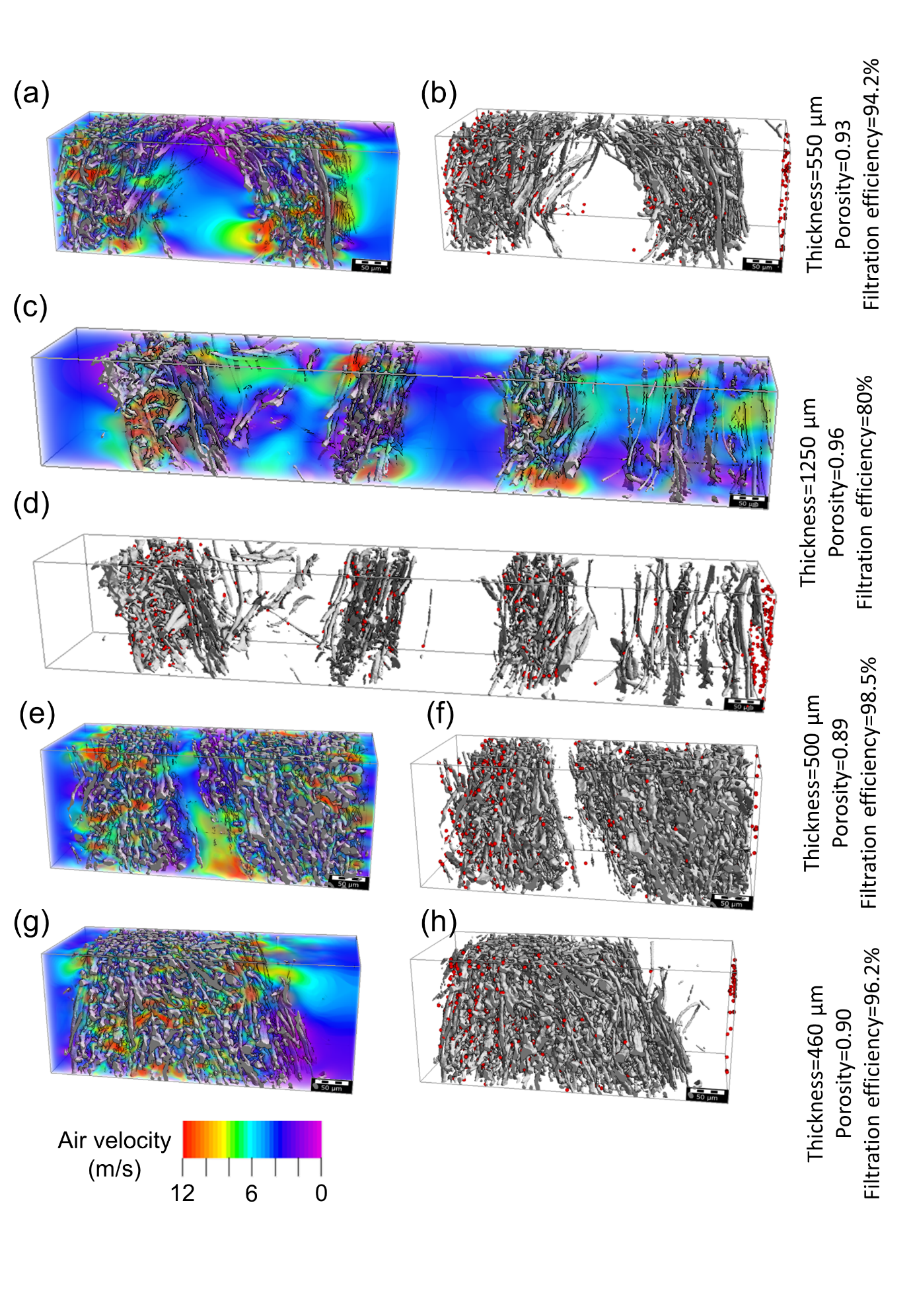


**Figure S2.** Flow field and distribution of captured particles for selected subregions of samples after one cycle of treatment. Here, the flow fields after one cycle of H_2_O_2_, soap, UV, and autoclave treatment are shown as (a,c,e, and g), respectively, and the corresponding distribution of captured particles is shown in (b,d,f, and h), respectively. The thickness, porosity, and filtration efficiency of the subregions chosen are mentioned alongside the corresponding flow field and distribution of captured particles.

**Figure S3.** Distribution of the penetration depth of 0.3 $\mu m$ particles for different selected sub-regions of melt-blown specimens (a) before, (b,d,f, and h) after one cycle, and (c,e,g, and i) after five cycles of H_2_O_2_, soap, UV, and autoclave decontamination treatments, respectively.
